# Editorial: Vascular co-option and beyond for cancer biology

**DOI:** 10.3389/fonc.2023.1227540

**Published:** 2023-06-19

**Authors:** Francesco Pezzella, Chao-Nan Qian

**Affiliations:** ^1^ Nuffield Division of Clinical Laboratory Science-Radcliffe Department of Medicine (NDCLS-RDM) John Radcliffe Hospital, University of Oxford, Oxford, United Kingdom; ^2^ Department of Radiation Oncology, Guangzhou Concord Cancer Center, Guangzhou, China; ^3^ Sun Yat-sen University Cancer Center, Guangzhou, China

**Keywords:** vessels, co-option, angiogenesis, non-angiogenic tumors, treatment

Since this Research Topic was launched, the third paper on the Hallmarks of Cancer has been published (1). In the first version, published in the year 2000, the authors wrote that “those researching the cancer problem will be practicing a dramatically different type of science than we have experienced over the past 25 years. Surely much of this change will be apparent at the technical level. But ultimately, the more fundamental change will be conceptual” (2), and this is reflected in the successive revised versions (1, 3). Among the conceptual changes that actually occurred, the paradigm switch summarized by the substitution of “inducing angiogenesis” with “inducing or accessing vasculature” is possibly the major one.

The original “Sustained angiogenesis” hallmark reflected the hypothesis that a tumor could grow beyond a few millimeters in diameter only if new vessels were formed to support it (4). This concept had a profound impact on the clinical research in oncology during the 1990s and the first decade of the 2000s as anti-angiogenic drugs were seen as not only a “universal” approach to cancer treatment (5) but also as a tumor therapy free from the drug resistance mechanisms (6). Furthermore, it was suggested that the number of micro-vessels in a neoplastic lesion was going to be one of the most important prognostic factors across different types of tumors (7) while, once again, things were not so straightforward (8).

The realization that tumors can actually grow also in absence of neo-angiogenesis, i.e., by co-opting previously existing normal vessels, has unveiled new unexpected aspects of cancer biology (9, 10). This is reflected by the two reviews and the hypothesis papers present in our Research Topic that examine the state of knowledge on vascular co-option in both tumor growth and tumor spreading (Cuypers et al., Ribatti et al., Lugassy et al.).

The existence of non-angiogenic tumors leads to the prediction that such a tumor would not be sensitive to current anti-angiogenic treatment (11) for self-explicative reasons. Subsequently, more causes of resistance to anti-angiogenic therapies have been discovered (12). To add insult to injury, it started to emerge that, actually, far from treating any type of cancers, anti-angiogenic therapy could, in some experimental models, actually increase tumor growth (13–16) with some safety issues raised for some groups of patients (17, 18).

One issue linked to these findings, and initially overlooked, is the actual effects of VEFG on the neoplastic cells, rather than on the vascular endothelium (19). This is the issue explored in this Research Topic by Liu et al., which further confirms that blocking VEGF can actually increase the growth capacity of a cancer cell and propose a possible mechanism. Bajbouj et al. describe how low levels of Vitamin D can cause, in some breast cancer cell lines, a similar aggressive non-angiogenic phenotype. In both papers, alterations in cell motility, adhesion, and cell proliferation are reported to occur with the involvement of some common pathway, e.g., TGF-beta and VEGF. These are findings very similar to that observed when looking at differences between angiogenic and non-angiogenic tumors, growing by vascular co-option (9). Eventually, both papers highlight the need for a better understanding of the VEGF and other angiogenic pathways and that caution is needed in therapeutic approaches as our knowledge of the underlying biology is still incomplete.

Given the failure of antiangiogenic treatments as single agents to uphold the expectations generated during the 1990s, it is only natural that the possibility of optimizing their use, mostly in combination therapy, has been increasingly explored. One of the most promising approaches is the combination with check point inhibitors aimed to preserve T-cell immunity function (20). Paulsen et al. report that a higher number of infiltrating CD8 positive T lymphocytes is associated with better outcome in squamous cell carcinoma. This result and the observation that the absolute number of CD8 positive T lymphocytes is particularly high in the diffuse subgroup of angiogenic tumors further support the rationale for associating anti-angiogenic and immune therapy.

However, not only the lymphocytes are involved, as a higher number of neutrophils in non-angiogenic, rather than angiogenic, colorectal metastases in the liver have been reported (21), and Rada et al. are looking into the molecular basis of this event. Their findings indicate that higher levels of RUNX1 in non-angiogenic metastatic cells activate the TGFbeta1 pathway, causing higher levels of Ang1 in the nearby normal hepatocytes, eventually leading to the migration of neutrophils in the metastatic lesions. RUNX1 is involved in many pathways including induction of angiogenesis (22); however, it would not be the first case of a protein involved in angiogenesis being involved in non-angiogenic malignancy as well (9). The main thing of course is to address the issue of these neutrophils, which could lead to possible new therapeutic tools in treating these other way very aggressive neoplastic lesions (21).

The aggressive nature of non-angiogenic malignancies is further examined by Paulsen et al. The authors confirm that full non-angiogenic lung adenocarcinoma has the worst outcome, but they also demonstrate that a non-angiogenic component in a predominantly angiogenic adenocarcinoma could also predict a worse prognosis.

It is therefore not by chance that most of the original papers in our Research Topic are about learning to exploit the interaction between cancer cells and vessels as possible new targets for treatment. As the second volume of this Research Topic is about to be launched, we very much look forward to new contributions on this complex issue.

## Author contributions

All authors listed have made a substantial, direct, and intellectual contribution to the work and approved it for publication.

## References

[1] Hanahan D. Hallmarks of cancer: new dimensions. Cancer Discov (2022) 12(1):31–46. doi: 10.1158/2159-8290.CD-21-1059 35022204

[2] Hanahan D Weinberg RA. The hallmarks of cancer. Cell (2000) 100(1):57–70. doi: 10.1016/S0092-8674(00)81683-9 10647931

[3] Hanahan D Weinberg RA. Hallmarks of cancer: the next generation. Cell (2011) 144(5):646–74. doi: 10.1016/j.cell.2011.02.013 21376230

[4] Folkman J. What is the evidence that tumors are angiogenesis dependent? J Natl Cancer Inst (1990) 82(1):4–6. doi: 10.1093/jnci/82.1.4 1688381

[5] Folkman J. Angiogenesis: an organizing principle for drug discovery? Nat Rev Drug Discov (2007) 6(4):273–86. doi: 10.1038/nrd2115 17396134

[6] Boehm T Folkman J Browder T O’Reilly MS. Antiangiogenic therapy of experimental cancer does not induce acquired drug resistance. Nature (1997) 390(6658):404–7. doi: 10.1038/37126 9389480

[7] Weidner N. Intratumor microvessel density as a prognostic factor in cancer. Am J Pathol (1995) 147(1):9–19. 7541613 PMC1869874

[8] Trivella M Pezzella F Pastorino U Harris AL Altman DG Prognosis In Lung Cancer Collaborative Study G. Microvessel density as a prognostic factor in non-small-cell lung carcinoma: a meta-analysis of individual patient data. Lancet Oncol (2007) 8(6):488–99. doi: 10.1016/S1470-2045(07)70145-6 17513172

[9] Donnem T Reynolds AR Kuczynski EA Gatter K Vermeulen PB Kerbel RS. Non-angiogenic tumours and their influence on cancer biology. Nat Rev Cancer (2018) 18(5):323–36. doi: 10.1038/nrc.2018.14 29520090

[10] Kuczynski E Vermeulen PB Pezzella F Kerbel RS Reynolds AR. Vessel co-option in cancer. Nat Rev Clin Oncol (2019) 16(8):469–93. doi: 10.1038/s41571-019-0181-9 30816337

[11] Pezzella F Pastorino U Tagliabue E Andreola S Sozzi G Gasparini G. Non-small-cell lung carcinoma tumor growth without morphological evidence of neo-angiogenesis. Am J Pathol (1997) 151(5):1417–23. PMC1858069 9358768

[12] Pezzella F. Mechanisms of resistance to anti-angiogenic treatments. Cancer Drug Resist (2019) 2(3):595–607. doi: 10.20517/cdr.2019.39 35582580 PMC8992538

[13] Lu KV Chang JP Parachoniak CA Pandika MM Aghi MK Meyronet D. VEGF inhibits tumor cell invasion and mesenchymal transition through a MET/VEGFR2 complex. Cancer Cell (2012) 22(1):21–35. doi: 10.1016/j.ccr.2012.05.037 22789536 PMC4068350

[14] Sennino B Ishiguro-Oonuma T Wei Y Naylor RM Williamson CW Bhagwandin V. Suppression of tumor invasion and metastasis by concurrent inhibition of c-met and VEGF signaling in pancreatic neuroendocrine tumors. Cancer Discov (2012) 2(3):270–87. doi: 10.1158/2159-8290.CD-11-0240 PMC3354652 22585997

[15] Kuczynski EA Yin M Bar-Zion A Lee CR Butz H Man S. Co-Option of liver vessels and not sprouting angiogenesis drives acquired sorafenib resistance in hepatocellular carcinoma. J Natl Cancer Inst (2016) 108(8). doi: 10.1093/jnci/djw030 PMC5017954 27059374

[16] Leenders WP Kusters B Verrijp K Maass C Wesseling P Heerschap A. Antiangiogenic therapy of cerebral melanoma metastases results in sustained tumor progression via vessel co-option. Clin Cancer Res (2004) 10(18 Pt 1):6222–30. doi: 10.1158/1078-0432.CCR-04-0823 15448011

[17] Kerr DJ Young AM. Targeted therapies: bevacizumab–has it reached its final resting place? Nat Rev Clin Oncol (2011) 8(4):195–6. doi: 10.1038/nrclinonc.2011.32 21386819

[18] Thompson EM Frenkel EP Neuwelt EA. The paradoxical effect of bevacizumab in the therapy of malignant gliomas. Neurology (2011) 76(1):87–93. doi: 10.1212/WNL.0b013e318204a3af 21205697 PMC3030223

[19] Goel HL Mercurio AM. VEGF targets the tumour cell. Nat Rev Cancer (2013) 13(12):871–82. doi: 10.1038/nrc3627 PMC4011842 24263190

[20] Yi M Jiao D Qin S Chu Q Wu K Li A. Synergistic effect of immune checkpoint blockade and anti-angiogenesis in cancer treatment. Mol Cancer (2019) 18(1):60. doi: 10.1186/s12943-019-0974-6 30925919 PMC6441150

[21] Palmieri V Lazaris A Mayer TZ Petrillo SK Alamri H Rada M. Neutrophils expressing lysyl oxidase-like 4 protein are present in colorectal cancer liver metastases resistant to anti-angiogenic therapy. J Pathol (2020) 251(2):213–23. doi: 10.1002/path.5449 32297656

[22] Lee SH Manandhar S Lee YM. Roles of RUNX in hypoxia-induced responses and angiogenesis. Adv Exp Med Biol (2017) 962:449–69. doi: 10.1007/978-981-10-3233-2_27 28299673

